# Positively and negatively charged additives modulate microbial-induced carbonate precipitation (MICP) for lead-contaminated loess remediation

**DOI:** 10.1039/d5ra06922j

**Published:** 2026-01-05

**Authors:** Jihua Gao, Wenle Hu, Pengli He, Longping Luo, Shixu Zhang, Zifeng Hui, Chongyang Zhang, Kangwei Wang, Rong Fan

**Affiliations:** a School of Architectural Engineering, HeNan Quality Polytechnic Pingdingshan 467000 China 659093786@qq.com; b Henan Key Laboratory of Green Building Materials Manufacturing and Intelligent Equipment, School of Intelligent Construction and Civil Engineering, Luoyang Institute of Science and Technology Luoyang 471023 China wenlehu@xauat.edu.cn; c School of Civil Engineering, Xi'an University of Architecture and Technology Xi'an 710055 China zhangsx@xauat.edu.cn; d School of Intelligent Construction and Civil Engineering, Luoyang Institute of Science and Technology Luoyang 471023 China hhzhly@lit.edu.cn 18337547198@163.com 16638536361@163.com 18238908195@163.com 17638348638@163.com; e School of Civil Engineering and Architecture, Xi'an University of Technology Xi'an 710048 Shaanxi China llp@xauat.edu.cn

## Abstract

The remediation of heavy metal-contaminated loess remains a critical environmental and geotechnical challenge. In this study, microbial-induced carbonate precipitation (MICP) was applied to Pb-contaminated loess with three representative additives: graphene oxide (GO), calcium lignosulfonate (Ca-Ls) and chitosan (CS). Mechanistic evaluation combined zeta potential analysis, scanning electron microscopy (SEM), unconfined compressive strength (UCS) tests and Pb^2+^ leaching experiments under freeze–thaw cycles. Results show that GO enhanced surface charge density, with zeta potential reaching about minus sixteen millivolts, contracted the diffuse double layer, and produced dense carbonate bridges. This treatment yielded the highest UCS, reaching about four hundred and sixty kilopascals initially and maintaining about three hundred and fifty kilopascals after nine freeze–thaw cycles. Pb^2+^ leaching in the GO group remained low, between fifty-five and sixty-eight milligrams per liter, corresponding to a reduction of about sixty-five percent compared with untreated loess. Ca-Ls achieved moderate improvements, retaining UCS at about three hundred and thirty kilopascals and restricting Pb^2+^ leaching to seventy to eighty milligrams per liter after cycling, consistent with uniform carbonate precipitation observed in SEM. In contrast, CS induced more negative potentials, about minus sixteen point two millivolts, but suppressed microbial activity, leading to patchy precipitation and higher leaching levels of ninety to ninety-five milligrams per liter. Collectively, the findings demonstrate that additive regulation of diffuse double layer characteristics and precipitation pathways governs both mechanical durability and heavy metal stabilization. GO provided the most favorable balance between strength and Pb immobilization, followed by Ca-Ls, while CS showed limited benefits. This study provides new insights into additive-assisted MICP as a practical and sustainable strategy for improving the environmental safety and engineering reliability of Pb-contaminated loess under freeze–thaw conditions.

## Abstract 2

Positively and negatively charged additives tune MICP to strengthen Pb-contaminated loess. The optimized system enhances carbonate precipitation, improves Pb immobilization, and increases soil mechanical performance *via* controlled nucleation and crystal growth.

## Graphical abstract

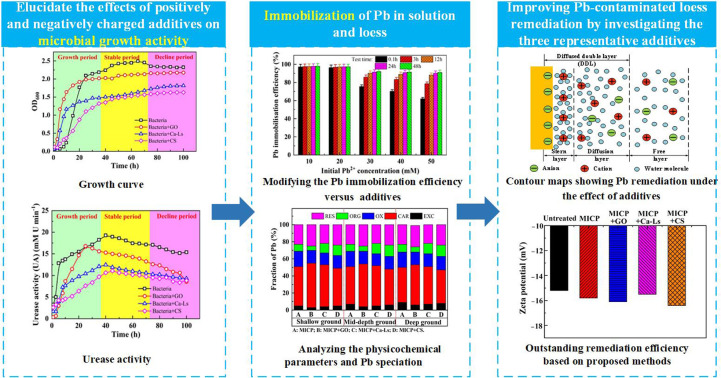

## 1. Introduction

Rapid industrialization and urban expansion in northwestern (NW) China have intensified environmental pollution, posing substantial risks to geotechnical infrastructure integrity, subsurface environmental quality, and public health.^1,2^ Among the principal contaminants, heavy metals such as copper (Cu) and lead (Pb), originating predominantly from mining, smelting, wastewater discharge, and the agricultural application of fertilizers and pesticides, migrate and accumulate within soil systems.^3,4^ This has resulted in widespread contamination of loess deposits, which are predominant in NW China and particularly vulnerable due to their unsaturated nature, structurally fragile fabric, and distinctive geotechnical properties.^5,6^ Nationwide surveys indicate that approximately 20% of arable land in China is affected by heavy metal pollution, with Pb concentrations in many regions exceeding the national Grade II standard for farmland environmental quality over the past two decades. Loess, characterized by high porosity, loose structure, and low water retention capacity, exhibits a markedly greater propensity for heavy metal accumulation and migration compared with other soil types.^7^ These inherent properties not only accelerate contaminant transport and buildup within the soil matrix but also complicate subsequent remediation efforts. Heavy metal contamination in loess regions is often associated with strong toxicity, high concealment, and long-term persistence,^8^ thereby exerting sustained threats to soil ecosystem stability and regional geotechnical safety, and attracting increasing attention in both environmental and engineering disciplines.^1,5,6^ In particular, remediation of loess under Pb remains technically challenging, as conventional physical, chemical, and biological methods often suffer from low efficiency, high costs, and risks of secondary pollution. This underscores the urgent need for efficient, cost-effective, and adaptable remediation technologies capable of simultaneously enhancing environmental quality and the engineering performance of heavy metal-contaminated loess, thereby supporting sustainable land reuse and effective risk management in vulnerable loess regions.

A wide portfolio of strategies have been applied for the remediation of heavy metal-contaminated soils, including phytoremediation,^9–12^ thermal desorption,^13–15^ soil replacement,^16^ stabilization/solidification,^17–19^ chemical leaching,^20,21^ and electrokinetic remediation technology.^22–24^ In recent years, microbial-induced calcium carbonate precipitation (MICP) has attracted increasing attention due to its environmental compatibility, operational simplicity, and potential for *in situ* applications. The fundamental principle of MICP relies on a series of complex biochemical reactions during microbial mineralization.^25–27^ Under favorable conditions, most microorganisms can, through metabolic activity and chemical reactions with cementation solutions, induce the precipitation of calcium carbonate (CaCO_3_), typically from mixtures of urea and calcium chloride.^28^ The primary focus of MICP-based techniques lies in promoting CaCO_3_ precipitation. Within the cementation solution, urea undergoes enzymatic hydrolysis catalyzed by urease, leading to carbonate ion generation, which subsequently reacts with calcium ions to form CaCO_3_. These characteristics have enabled MICP technology to find broad applications across geotechnical engineering, construction materials, and environmental engineering. Over years of research and practical implementation, scholars have increasingly explored the use of various additives to optimize MICP performance, thereby achieving notable improvements in diverse engineering contexts. For example, Hanisch *et al.*^29^ investigated the effects of different additives, including calcium bentonite, sodium bentonite, clinoptilolite, sodium zeolite, limestone, marl clay, cresol, thioether, and activated carbon powder, on the solidification performance of MICP in sand. Their results demonstrated that these additives influenced the adsorption of ureolytic bacteria and the fixation of ammonium, with calcium bentonite and clinoptilolite showing the most pronounced enhancement. He *et al.*^30^ examined six inorganic additives including zeolite, bentonite, sodium metasilicate, sodium metaaluminate, gypsum, and quicklime (CaO), and found that CaO was the most effective additive, with an optimal dosage of 5%. Under the synergistic action of MICP and CaO, a large amount of calcite was generated, binding tailings particles together while incorporating heavy metal ions into the calcite lattice. Duan *et al.*^31^ proposed the integration of montmorillonite with MICP (Mt-MICP) for the stabilization of cyanide tailings. Their findings indicated that Mt-MICP markedly increased mineral precipitation, reduced both total and free cyanide concentrations, and lowered the leaching levels of Cr, Zn, Cu, and Pb. Moreover, montmorillonite enhanced the uniformity of biocementation and improved the CO_2_ sequestration capacity of tailings. Density functional theory (DFT) and thermogravimetric (TG) analyses further confirmed that montmorillonite increased the adsorption energy of tailings for CO_2_ and promoted the urease-catalyzed transformation of urea into CaCO_3_. In addition to these studies, other additives investigated for MICP applications include discarded mask fibers,^32^ xanthan gum,^33^ volcanic ash,^34^ graphene oxide (GO),^35^*N*-butyl-thiophosphoryl triamide,^36^ and superabsorbent polymers.^37^ However, the majority of these applications have primarily emphasized the ultimate treatment outcomes, while comparatively little attention has been given to the effects of additives on the underlying MICP reaction processes.^38–40^ Since such effects are crucial determinants of overall treatment efficiency, understanding additive–MICP interactions at the mechanistic level remains a significant research gap. Studies have shown that the inclusion of additives such as serum albumin, biofilm surface proteins, magnesium chloride, and poly-l-lysine can significantly influence the crystallization process.^41–44^ Compared with additive-free MICP, reactions incorporating most additives yield substantially higher calcite content after 24 h, except for magnesium chloride, which favors the formation of magnesian calcite.^45^ Furthermore, doping with biofilm surface proteins results in larger crystal sizes relative to other additives. These findings indicate that additives can modify both the morphology and dimensions of CaCO_3_ crystals in MICP. The precipitation process is driven by the interaction between negatively charged ions released from microbial surfaces and CO_3_^2−^ and Ca^2+^ ions in solution, highlighting that the interplay between microbial surface charge and bacterial activity constitutes a key mechanistic pathway in MICP technology.

These studies collectively suggest that the introduction of different types of additives can provide deeper insights into the charge interactions occurring in MICP reactions, thereby allowing inferences about the stability and mechanisms of different reaction systems.^46^ To date, however, most MICP research has concentrated on the kinetics of mineralization and the mechanisms of soil reinforcement. What remains lacking is a mechanistic understanding of how additives influence MICP processes from the perspective of positive and negative charge interactions, and how such mechanisms govern the remediation of Pb-contaminated loess. This knowledge gap limits the effective utilization of MICP technology. To address this issue, the present study focuses on the role of charge characteristics in additive solutions. Specifically, chitosan was employed as a positively charged additive, while graphene oxide (GO) and calcium lignosulfonate were selected as negatively charged additives. By systematically comparing these additives, we investigated their effects on microbial growth activity, the efficiency of MICP reactions, and the mechanical and toxicity-related properties of Pb-contaminated loess. These preliminary findings provide theoretical guidance for advancing the application of MICP in real-world engineering contexts. Nevertheless, the use of additives inevitably increases the complexity and cost of MICP remediation, which remains a limiting factor in its large-scale deployment. Furthermore, several promising research directions warrant further exploration. These include broadening the range of commonly used additives with different charge types and examining how CaCO_3_ produced under microwave plasma treatment of additives influences its elemental composition and structural characteristics. Such investigations will help to evaluate the applicability of microwave plasma-assisted MICP across diverse contaminated sites. Taken together, these efforts are expected to provide more comprehensive theoretical support for enhancing the effectiveness and practical application of MICP in heavy metal-contaminated loess remediation.

Despite these advances, most studies emphasize end-point metrics (*e.g.*, unconfined compressive strength, permeability, metal leachability), while comparatively little attention has been paid to how additives regulate the reaction pathway of MICP—namely, bacterial activity, interfacial charge, nucleation kinetics, and crystal evolution.^38,39^ Yet the MICP microenvironment is inherently electrostatic: bacterial envelopes and extracellular polymers bear charges; solution speciation controls the activities of CO_3_^2−^ and Ca^2+^; and charged macromolecules or nanomaterials can reshape double-layer interactions, aggregation, and ion transport. Indeed, proteins, polyelectrolytes, Mg^2+^, and biofilm components have been shown to alter polymorph selection, crystal size, and morphology—often increasing calcite yield within 24 h or, in Mg-rich systems, shifting to magnesian calcite.^41–45^ These observations point to charge-mediated control of CaCO_3_ precipitation and, by extension, of metal co-precipitation, adsorption, and occlusion. In light of this, the objectives of this study are to: (1) elucidate the effects of positively and negatively charged additives on microbial growth activity and the overall efficiency of MICP reactions in Pb-contaminated loess; (2) evaluate the influence of different additives on the mechanical properties, toxicity reduction, and remediation performance of Pb-contaminated loess under MICP treatment; (3) reveal the underlying mechanisms of charge interactions in MICP systems and provide theoretical guidance for the practical application of additive-assisted MICP in heavy metal-contaminated soils.

## 2. Materials and methods

### 2.1 Specimen preparation

**Fig. 1:**
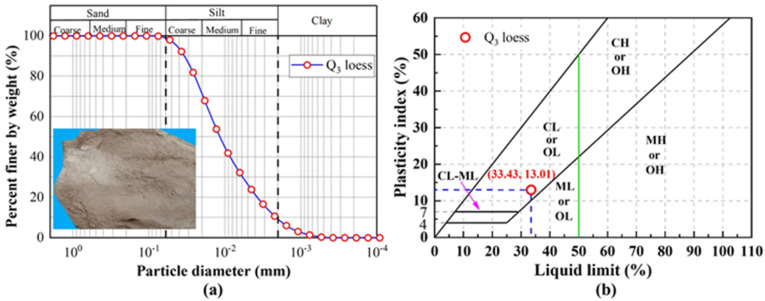
(a) Particle size distribution of loess and (b) liquid limit and plastic index.

The loess samples used in this study were collected from the vicinity of Tongchuan City, Shaanxi Province, located within the structural unit of the Guanzhong Basin in the middle reaches of the Yellow River.^1,2^ From a regional geological perspective, the Guanzhong area represents a core component of the Loess Plateau. The loess deposits covering this region constitute the largest and most continuous Quaternary aeolian loess sequence worldwide, with a geographical distribution of approximately 6.4 × 10 ^5^ km^2^. These deposits not only preserve critical records of global climate change and regional environmental evolution since the Late Pleistocene but also serve as a representative medium for studies on the mechanical behavior of loess, its engineering geological properties, and geochemical cycles. Consequently, they provide an ideal natural material for investigating the mechanisms of geological hazards in the Loess Plateau and for evaluating the suitability of loess areas for engineering construction. Disturbed material was sampled from 0.5 to 1.0 m depth, sealed in the field, and transferred to the laboratory. As collected, the sediment was unconsolidated and pale grey-yellow. Pre-treatment followed the protocols of Xu *et al.*^6^ Grain-size distributions were measured by laser-diffraction granulometry (Mastersizer 2000; Malvern Panalytical). Under the Unified Soil Classification System (ASTM, 2011),^57^ the material is classified as CL (low-plasticity clay), in agreement with the United States Department of Agriculture textural scheme (1951). Index physical–mechanical properties are listed in Fig. 1 and Table 1. Bulk geochemistry determined by inductively coupled plasma mass spectrometry (ICP-MS) shows SiO_2_ as the predominant oxide, followed by Al_2_O_3_, CaO and Fe_2_O_3_.

**Table 1:** Physicochemical properties of the loess

Physical index	Data
Fines (%)	94.16
Sand (%)	5.84
Gravel (%)	0
Specific gravity, gs	2.70
Void ratio, e	0.86
Dry density, *ρ*_dmax_/(g cm^−3^)	1.73
Initial water content, (%)	16.6
The atterberg limit	
Liquid limit, (%)	33.43
Plastic limit, (%)	13.01
Soil classification	CL

### 2.2 Bacteria and cementation solutions for MICP treatment

MICP relies on ureolytic, alkaliphilic microorganisms that hydrolyze urea under alkaline conditions, producing carbonate ions that react with Ca^2+^ to precipitate CaCO_3_. The crystals fill pores and form interparticle cementation bridges, enhancing soil strength, stiffness, and structural integrity while reducing permeability. At the microscale, carboxyl and phosphate groups on cell walls and extracellular polymeric substances provide negatively charged nucleation sites that promote the preferential growth of CaCO_3_ polymorphs such as calcite, vaterite, and aragonite. The nucleation pathway and crystal morphology are regulated by supersaturation, ionic strength, pH, temperature, and organic or impurity ions, ultimately controlling the mechanical and hydraulic behavior of treated soils.

Beyond soil reinforcement, MICP can immobilize heavy metals through co-precipitation, lattice substitution, and surface adsorption–encapsulation, thereby reducing their mobility and bioavailability. Among functional microbes, Sporosarcina pasteurii has emerged as the most effective ureolytic bacterium due to its high enzyme activity, environmental adaptability, and ease of cultivation. In this study, S. pasteurii (CGMCC 1.3687) was selected as the inoculum. The culture medium, modified from Jiang *et al.*^47^ and Wang *et al.*,^48^ contained urea (20 g L^−1^) as the substrate, peptone (5 g L^−1^) and yeast extract (3 g L^−1^) as nutrient sources, and manganese sulfate (0.01 g L^−1^) as a trace element. The medium was adjusted to pH 7.0 with 10% NaOH, sterilized at 121 °C for 20 min, cooled, and inoculated.

### 2.3 Preparation of positive and negative charge additives

Chitosan (CS) is a cationic polysaccharide whose free amino groups (–NH_2_) are readily protonated to –NH_3_^+^ under acidic conditions, thereby imparting positive charge. Previous studies have shown that CS can interact electrostatically with negatively charged groups on cell surfaces or extracellular polymeric substances, improving microbial dispersion and providing nucleation sites for heterogeneous CaCO_3_ precipitation, while also helping to maintain or enhance urease activity within a certain range, thus facilitating MICP-induced cementation.^49–51^ In this study, analytical-grade CS (Sinopharm Group) was selected as the cationic additive. The preparation procedure was as follows: an appropriate amount of CS powder was gradually added into a pre-prepared aqueous acetic acid solution (0.5% v/v) and magnetically stirred at 50–60 °C until completely dissolved, yielding a CS solution with a target concentration of 5 g L^−1^.^52^ After cooling to room temperature, the pH was carefully adjusted to 6.8 using 1 M NaOH to avoid localized alkalinity that could cause CS precipitation. If necessary, insoluble residues were removed using a 0.45 µm microporous membrane filter. The resulting solution was stored in the dark at 4 °C and used within 7 days.

Graphene oxide (GO) is a widely studied two-dimensional nanomaterial whose surface is enriched with oxygen-containing functional groups, including carboxyl, hydroxyl, and epoxy moieties. These groups impart pronounced negative charge and strong adsorption/complexation capacity.^53–55^ In addition, they can act as heterogeneous nucleation sites for CaCO_3_ and regulate crystal nucleation and morphology through a “surface templating effect.” In this study, GO supplied by Xi'an University of Architecture and Technology (thickness 4–5 nm, lateral size 0.5–3.5 µm) was employed. A stock suspension with a target concentration of 10 mg L^−1^ was prepared by dispersing GO powder into ultrapure water, followed by ultrasonic treatment (intermittent sonication under ice-bath conditions to avoid overheating and sheet fragmentation) until a stable dispersion was obtained. When necessary, a trace amount of inert dispersant (non-interfering with microbial activity or cementation chemistry) was added to further enhance suspension stability. The dispersion was filtered through a 0.45 µm membrane to remove large aggregates, stored in amber glass bottles at 4 °C in the dark, and used within 7 days.

Calcium lignosulfonate (Ca-Ls), a by-product of the sulfite pulping process, is an anionic natural polymer rich in sulfonate (–SO_3_^−^) and carboxylate (–COO^−^) functional groups. Its polyanionic nature imparts strong dispersing, chelating, and water-reducing properties. On one hand, Ca-Ls can form complexes or bridging interactions with Ca^2+^ and heavy metal ions, thereby influencing their mobility and effective activity in pore water. On the other hand, it acts as a “soft template” and crystal growth modifier, altering the nucleation kinetics, polymorphic distribution (for example, promoting or inhibiting the formation of calcite or aragonite), and morphology of CaCO_3_. In addition, the dispersing effect of Ca-Ls helps suppress premature local clogging and improves the spatial uniformity of MICP-induced cementation. For solution preparation, commercially available Ca-Ls (analytical grade or equivalent) was dissolved in ultrapure water to prepare a stock solution (1 g L^−1^ in this study). The solution was stirred until fully dissolved, and its pH was adjusted to 6.8 ± 0.1 using NaOH or HCl. If necessary, insoluble residues were removed by 0.45 µm filtration. The stock solution was stored at 4 °C in the dark and used within 7 days. All experiments reported in this work included three replicates, and statistical analysis showed that the coefficient of variation (COV) remained well below 10%, confirming the reproducibility of the data.

**Fig. 2:**
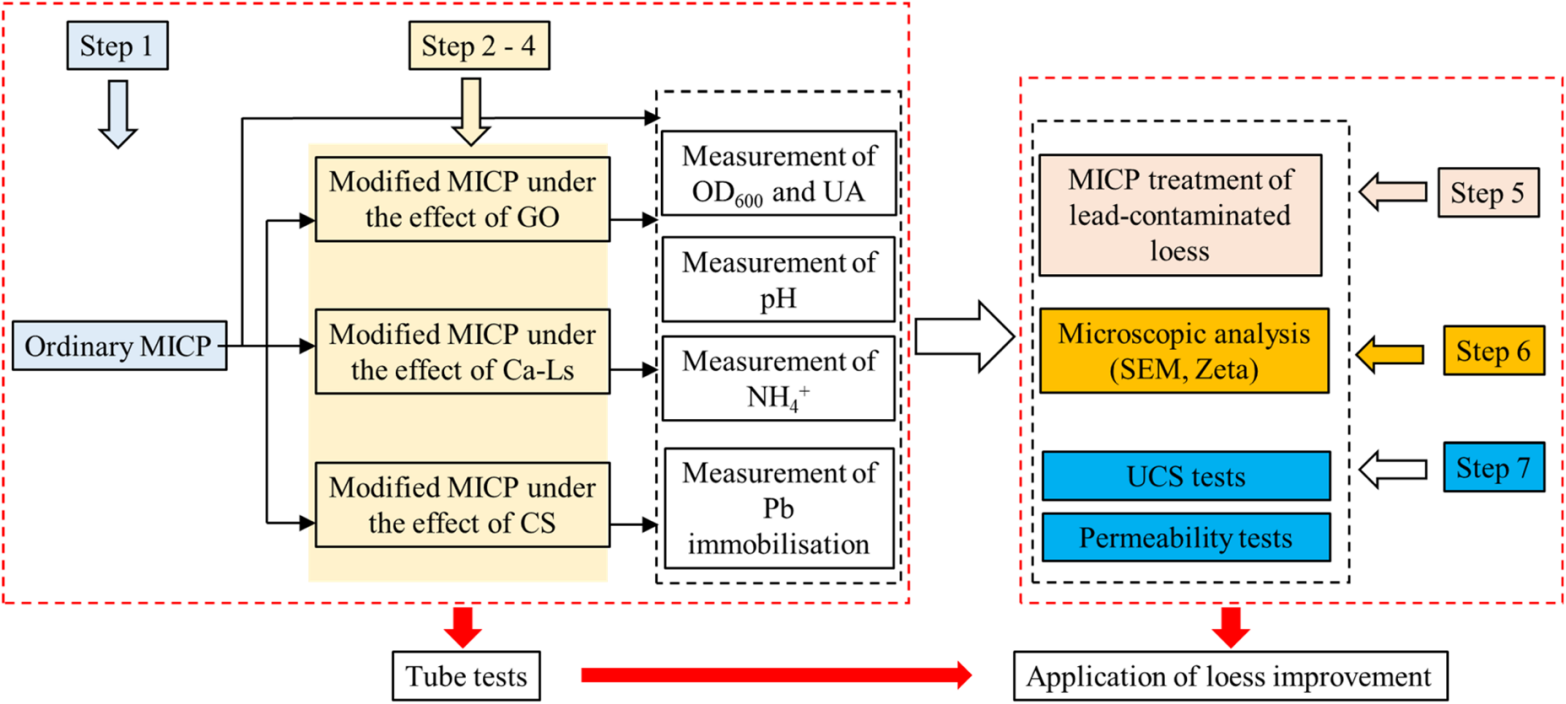
The procedures for experimental methods.

Accordingly, this study selected chitosan as a positively charged additive, and graphene oxide (GO) and calcium lignosulfonate (Ca-Ls) as negatively charged additives for comparison. These three representative additives embody distinct charge characteristics and surface chemistries, enabling a systematic evaluation of their roles within the MICP system in terms of: (i) their influence on microbial activity and spatial distribution; (ii) their regulation of carbonate nucleation processes; and (iii) their combined effects on macroscopic cementation strength and the stabilization of Pb in contaminated loess (see Fig. 2).

### 2.4 Testing of MICP under different additive conditions

#### 2.4.1 Bacterial cultivation and chemicals

The ureolytic bacterium Sporosarcina pasteurii was obtained from the China General Microbiological Culture Collection Center (CGMCC) and used as the functional strain in this study. The culture medium was composed of 20 g L^−1^ yeast extract, 10 g per L NH_4_Cl, 10 mg per L MnSO_4_·H_2_O, and 24 mg per L NiCl_2_·6H_2_O. A 0.1 mL aliquot of frozen bacterial stock was inoculated into 100 mL of medium and incubated at 30.0 °C and 180.0 rpm for 120 h. Growth curves and urease activity (UA) measurements indicated that the culture remained in the lag phase during the first 10 h, entered the logarithmic growth phase after 20 h, and reached the stationary phase after approximately 30 h. Accordingly, 30 h was considered sufficient for ureolytic bacterial growth and reproduction to achieve enhanced urea hydrolysis. All culture vessels were sterilized by autoclaving for 30 min before use, and the entire cultivation process was conducted under aseptic conditions.

#### 2.4.2 Immobilization Pb in solution

A Pb stock solution was prepared by dissolving Pb(NO_3_)_2_ (China Damao Chemical Reagent Co., Ltd) in distilled water, followed by filtration through a 0.22 µm membrane and storage at 4 °C. In this study, bacterial suspensions were transferred into 30.0 mL test solutions containing urea and 10–50 mM Pb^2+^, diluted with distilled water to a final volume of 60 mL. The control group consisted of sterile deionized water in place of the bacterial suspension. All treatments were incubated at 30 °C for 48 h. Concentrations of NH_4_^+^ and Pb^2+^ at 0, 24, and 48 h were quantified using a visible spectrophotometer (721G; Inesa Analytical Instrument, China) and an atomic absorption spectrophotometer (TAS-990; Puxi Universal Instruments, Beijing, China).

NH_4_^+^ concentrations were determined by the Nessler reagent colorimetric method. A calibration curve was established prior to measurement, and absorbance was recorded 10 min after adding Nessler's reagent and potassium sodium tartrate, with concentrations calculated from the calibration curve. For Pb^2+^ quantification, mixed solutions containing urease, urea, Ca^2+^, and Pb^2+^ were centrifuged at 10 000 rpm for 10 min, and the Pb^2+^ concentration in the supernatant was measured to determine immobilization efficiency.

#### 2.4.3 Specimen preparation

Pb-contaminated loess was prepared by mixing Pb stock solution, loess, and distilled water to achieve a target water content of 18% and a Pb^2+^ concentration of 3000 mg kg^−1^. The contaminated soil was sealed and stored in a humidity chamber for 72 h prior to specimen preparation. Cylindrical molds made of acrylic (height 80 mm) were used, with filter paper and gravel placed at both ends to ensure drainage. A total of 139.3 g of Pb-contaminated soil was divided into three layers. Each layer was compacted and lightly scarified before the next was added to prevent interfacial separation. Finally, cylindrical loess specimens with a diameter of 39.8 mm and a dry density of 1.4 g cm^−3^ were prepared for subsequent Pb immobilization tests.

#### 2.4.4 Immobilization Pb in loess soil

During the experiment, a peristaltic pump was used to inject the biogrout (bacterial/urease suspension) into the Pb-contaminated loess specimens at a flow rate of 5 mL min^−1^. The specimens were then cured for 1 h to allow the bacteria and urease to distribute uniformly throughout the soil matrix. Subsequently, the cementation solution (urea solution) was injected at the same flow rate, and the biomineralization reaction was allowed to proceed for 24 h. For analysis, soil samples were collected from three depth intervals: shallow (0–25 mm), middle (25–50 mm), and deep (50–75 mm).

### 2.5 Specimens characterization

#### 2.5.1 Physical and chemical performance tests

The biomineralization process can alter the surrounding pH, thereby influencing the speciation of heavy metals in soil. Accordingly, soil pH after biogrouting treatment was measured following the Standard for Soil Test Methods. Briefly, 10 g of oven-dried soil (passed through a 2 mm sieve) was placed in a 100 mL Erlenmeyer flask, and 50 mL of CO_2_-free distilled water was added to prepare a soil suspension at a soil-to-water ratio of 1 : 5. The suspension was shaken for 3 min and then allowed to stand for 30 min. The pH of the supernatant was measured using a benchtop pH meter, representing the acidity–alkalinity environment of the treated soil.

The speciation of heavy metals is a key indicator of their stability and ecological toxicity. Following biogrouting treatment, the chemical forms of Pb can undergo significant transformation, which in turn influences remediation efficiency. In this study, the speciation of Pb in soil was analyzed using the Tessier sequential extraction procedure, which partitions metals into five operationally defined fractions: exchangeable, carbonate-bound, Fe–Mn oxide-bound, organically bound, and residual. For each test, 1 g of oven-dried soil (sieved to 0.075 mm) was subjected to sequential extraction. The resulting solutions were filtered, diluted as required, and analyzed for Pb^2+^ concentration using atomic absorption spectrophotometry, thereby characterizing the distribution of Pb among different geochemical fractions.

#### 2.5.2 UCS tests

Unconfined compressive strength (UCS) tests were conducted to evaluate the mechanical performance of Pb-contaminated loess following MICP treatment. Cylindrical specimens with a diameter of 39.8 mm and a height of 80 mm were prepared as described in Section 2.4. After treatment and curing, the specimens were carefully demolded and trimmed to ensure smooth and parallel loading surfaces. UCS testing was performed using a universal testing machine (Instron 3369; Instron, USA) at a constant axial strain rate of 1 mm min^−1^ until failure. During each test, axial load and displacement were continuously recorded, and stress–strain curves were generated. The peak axial stress was defined as the UCS value.

#### 2.5.3 Leaching tests

The leaching behavior of Pb in solidified loess was evaluated using the U.S. EPA Toxicity Characteristic Leaching Procedure (TCLP, Method 1311),^56,57^ a widely adopted protocol for assessing the environmental risk of stabilized wastes. The extraction fluid was prepared by mixing 5.7 mL glacial acetic acid and 64.3 mL NaOH solution (1 N), diluted to 1 L with deionized water, and adjusted to pH 4.93 ± 0.05. Treated soil samples were air-dried, sieved to <2 mm, and 50 g subsamples were placed in 500 mL extraction vessels with 500 mL of leaching solution. The sealed vessels were agitated on a rotary shaker at 30 ± 2 rpm for 18 h. Supernatants were filtered through 0.45 µm membranes, and Pb concentrations were quantified using a polarized Zeeman atomic absorption spectrometer (Z-8200, Hitachi).

#### 2.5.4 Zeta potential tests

To investigate the influence of different additives on the surface charge characteristics of MICP-treated Pb-contaminated loess, zeta potential measurements were performed on loess samples solidified with (i) chitosan (CS), (ii) graphene oxide (GO), (iii) calcium lignosulfonate (Ca-Ls), and (iv) control specimens without additives. All samples were first air-dried to constant weight, ground, and passed through a 2 mm sieve. A suspension was prepared by dispersing 0.1 g of soil in 100 mL of deionized water (0.1 wt%). The suspensions were stirred magnetically for 30 min and subsequently subjected to ultrasonic treatment for 10 min to ensure homogeneity and minimize particle aggregation. For GO and Ca-Ls amended specimens, dispersion was further stabilized by intermittent ultrasonication prior to testing. By comparing zeta potential across the different additive treatments, this study aimed to elucidate how cationic (CS) and anionic (GO, Ca-Ls) additives alter the electrochemical surface properties of MICP-solidified Pb-contaminated loess, and to link these variations to mechanisms of carbonate precipitation and Pb immobilization.

#### 2.5.5 SEM tests

SEM was used to characterize microscale structural changes in Pb-contaminated loess before and after treatment. Particle morphology and interparticle bonding were examined using a Zeiss Gemini Sigma 300 (Oberkochen, Germany). The procedures followed established protocols with modifications from Xu *et al.*,^6^ He *et al.*,^58^ and Hou *et al.*^59^

## 3. Results and discussion

### 3.1 Effect of positive and negative charge additives on MICP

**Fig. 3:**
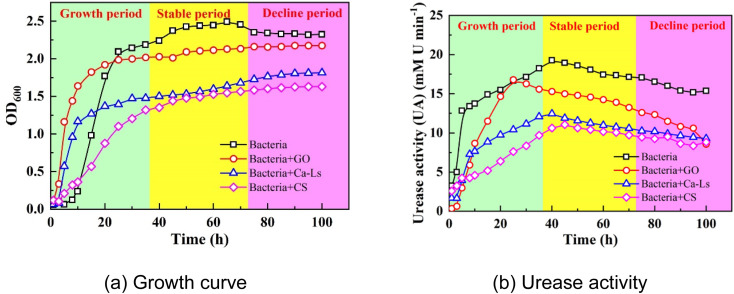
(a) Growth curve and (b) urease activity of MICP.

During culture preparation, GO, Ca-Ls, and CS were added separately along with bacterial inoculation, and growth and urease activity were monitored over 100 h. The resulting curves are shown in Fig. 3. Growth data indicated three distinct phases: an exponential phase characterized by rapid proliferation, a stationary phase with negligible growth, and a decline phase associated with cell lysis. The observed growth dynamics were consistent with previous reports, confirming the validity of the cultivation protocol used in this study. As shown in Fig. 3, bacterial cultures without additives exhibited the highest cell density and urease activity, outperforming those supplemented with CS, GO, or Ca-Ls. Among the treatments, CS and Ca-Ls produced the lowest values, followed by GO. These results suggest that positively charged additives exert stronger inhibitory effects on microbial growth and activity than negatively charged ones. Furthermore, the higher cell density and urease activity observed with Ca-Ls relative to CS indicate that a greater density of negative charges facilitates microbial growth by adsorbing Pb^2+^ ions and mitigating their toxicity.

### 3.2 Immobilization of Pb in solution

**Fig. 4:**
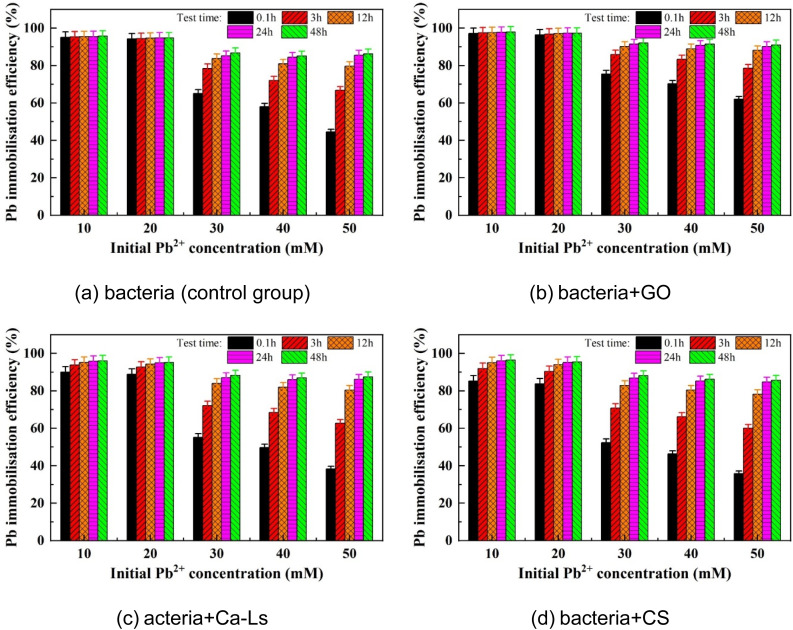
Relationships of Pb immobilization efficiency *versus* initial Pb^2+^ concentration: (a) bacteria (control group), (b) bacteria + GO, (c) bacteria + Ca-Ls and (d) bacteria + CS.

As shown in Fig. 4, systems with initial Pb^2+^ concentrations of 10–20 mM maintained nearly constant immobilization efficiency at 0.1 h, demonstrating early robustness under low to moderate metal loadings. By contrast, efficiencies declined sharply at 30–50 mM, with the steepest drop at 50 mM. Over 3–12 h, immobilization efficiency increased across all treatments, although high-concentration groups remained consistently lower than low-concentration ones. By 24 h, differences between treatments converged markedly, and values at 48 h showed little change, indicating stabilization of the system.

This temporal pattern reflects two main constraints. First, elevated Pb^2+^ loadings exert toxic effects on microbial activity and urease function, reducing the urea hydrolysis rate and delaying carbonate supersaturation.^25^ Second, in the early stages, carbonate availability and heterogeneous nucleation sites (*e.g.*, cell surfaces, EPS, mineral interfaces) are limited. Under high metal demand, this mismatch suppresses effective Pb–carbonate precipitation. With time, the formation of CaCO_3_ and Pb–carbonates increases surface coverage and induces local mass-transfer limitations, shifting the process toward a plateau phase.

It is important to note that the decline in immobilization efficiency (%) with increasing Pb^2+^ concentration does not necessarily indicate lower absolute immobilization. High-concentration groups may achieve greater absolute Pb removal, but larger denominators yield lower percentage values. Hence, both absolute immobilization and efficiency should be reported to avoid misinterpretation. Overall, alkaline conditions promote ureolytic bacterial growth, enhance carbonate precipitation, and reduce heavy metal bioavailability,^50,51^ explaining the observed time-dependent increases and convergence by 24–48 h.

### 3.3 Immobilization of Pb in loess

**Fig. 5:**
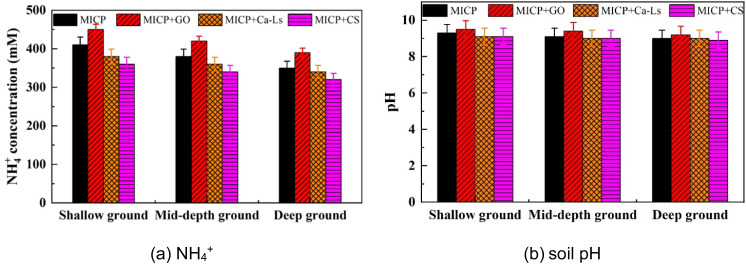
(a) Variation of NH_4_^+^ concentration and soil pH against Pb^2+^ concentration for the specimens taken from three depths considering different positive and negative charge additives: (a) NH_4_^+^ and (b) soil pH.

**Fig. 6:**
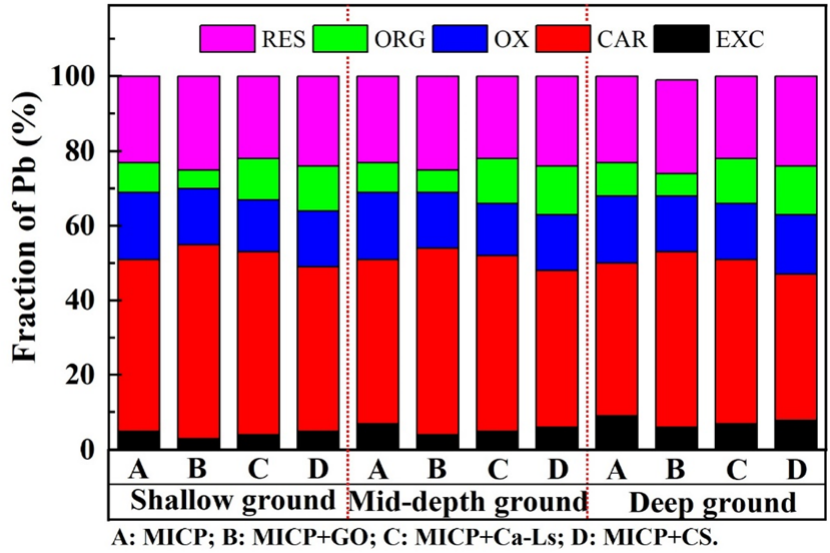
Variation of Pb species against Pb^2+^ concentration for the specimens taken from three depths considering different positive and negative charge additives.

As shown in Fig. 5a–b and 6, urea hydrolysis exhibited a clear dependence on both soil depth and additive type. Using NH_4_^+^ concentration as an indicator, the shallow specimens recorded approximately 450 mM for MICP + GO, about 415 mM for the control, approximately 395 mM for MICP + Ca-Ls, and about 370 mM for MICP + CS. At mid-depth, the concentrations were approximately 420, 385, 360, and 345 mM, respectively, while in the deep specimens they decreased further to about 395, 360, 340, and 330 mM. Compared with the control, GO increased NH_4_^+^ concentrations by about 8 percent across all depths, Ca-Ls reduced them by about 5 percent, and CS reduced them by 10 to 12 percent. From shallow to deep layers, NH_4_^+^ concentrations declined by 6 to 15 percent, indicating that negatively charged additives promoted urea hydrolysis more effectively than positively charged additives, while deeper layers were constrained by limited mass transfer, microbial distribution, and the availability of nucleation sites.

pH profiles further demonstrated that all treatments maintained a strongly alkaline environment conducive to ureolysis and carbonate precipitation. GO-treated samples showed the highest values (9.6–9.7), followed by the control (9.3–9.4), Ca-Ls (9.2–9.3), and CS (9.0–9.1). Depth effects were negligible (<0.1 pH units), suggesting robust alkaline buffering across layers, while additive type exerted a stronger control on microenvironmental conditions.

Sequential extraction results established a direct link between these physicochemical parameters and Pb speciation. Across all depths, Pb was dominated by carbonate-bound (48–52%) and residual (22–25%) fractions, while Fe–Mn oxide-bound (12–15%), organic-bound (10–12%), and exchangeable (5–8%) forms were markedly reduced. Additive-specific differences were evident: GO yielded the highest combined proportion of carbonate-bound and residual Pb (2–3% higher than the control) and the lowest exchangeable fraction, confirming superior stabilization. Ca-Ls was intermediate, while CS retained 1–2% more exchangeable Pb and correspondingly less carbonate-bound Pb, indicating weaker stabilization. Depth-related differences were modest (<3%), though shallow samples consistently exhibited slightly higher stable fractions, consistent with enhanced NH_4_^+^ generation and more active carbonate precipitation near the surface.

Mechanistically, the negatively charged functional groups of GO and Ca-Ls promote Pb^2+^ adsorption and serve as heterogeneous nucleation templates, thereby enhancing local carbonate supersaturation and nucleation probability. This is reflected in higher NH_4_^+^ levels, sustained alkalinity, and a greater proportion of carbonate-bound and residual Pb. In contrast, the cationic nature of CS may compete with microbial and EPS surface sites, suppressing cell dispersion and urease activity, resulting in lower NH_4_^+^ concentrations, reduced pH, and higher persistence of exchangeable Pb. Collectively, these findings demonstrate that additive-mediated regulation of microbial activity, porewater chemistry, and nucleation pathways underpins the observed differences in stabilization efficiency, while depth effects are primarily controlled by transport and distribution constraints. The quantitative evidence establishes a consistent causal chain: higher urea hydrolysis and stable alkaline conditions promote the transformation of Pb from labile to carbonate-bound and residual forms, thereby enhancing immobilization efficiency and long-term environmental safety.

### 3.4 Remediation of lead-contaminated loess

**Fig. 7:**
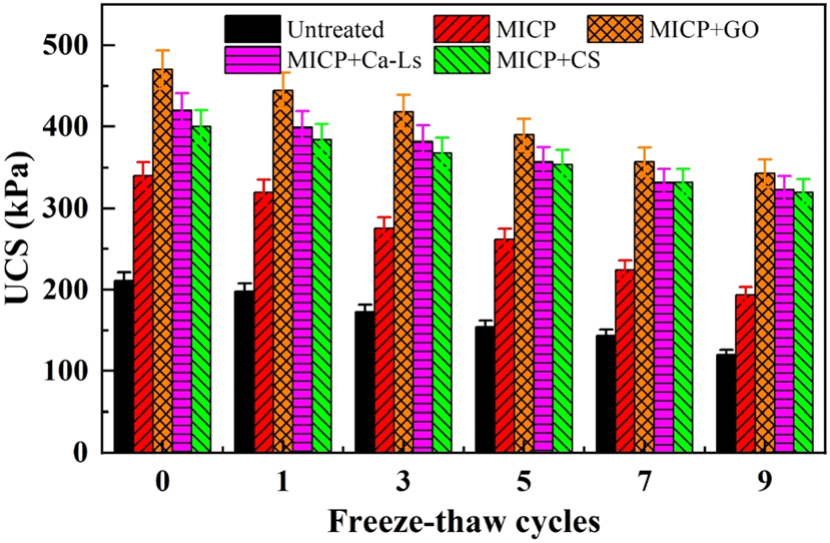
Relationship between unconfined compressive strength and additives under freeze–thaw cycles.

The effects of different additives on the unconfined compressive strength (UCS) and Pb^2+^ leaching behavior of MICP-stabilized loess were strongly modulated by freeze–thaw cycling (see Fig. 7). Before freezing, the UCS followed the order MICP + GO (approximately 460 kPa) > MICP + Ca-Ls (about 425 kPa) > MICP + CS (approximately 390 kPa) > MICP alone (about 340 kPa) > untreated loess (about 210 kPa). Strength declined progressively with increasing freeze–thaw cycles, yet the relative ranking remained unchanged. After nine cycles, UCS values decreased to about 350 kPa in the GO group, 330 kPa in the Ca-Ls group, 320 kPa in the CS group, 200–210 kPa in the MICP group, and 130 kPa in the untreated samples. In terms of retention, the Ca-Ls and GO groups preserved approximately 76–78 percent of their initial strength, the CS group retained about 82 percent, while the MICP and untreated groups maintained only 60–62 percent. These results indicate that while CS provided the highest relative retention, GO and Ca-Ls delivered both higher initial strengths and greater absolute durability compared with MICP alone, whose strength decayed at an average rate of about 15 kPa per cycle.

**Fig. 8:**
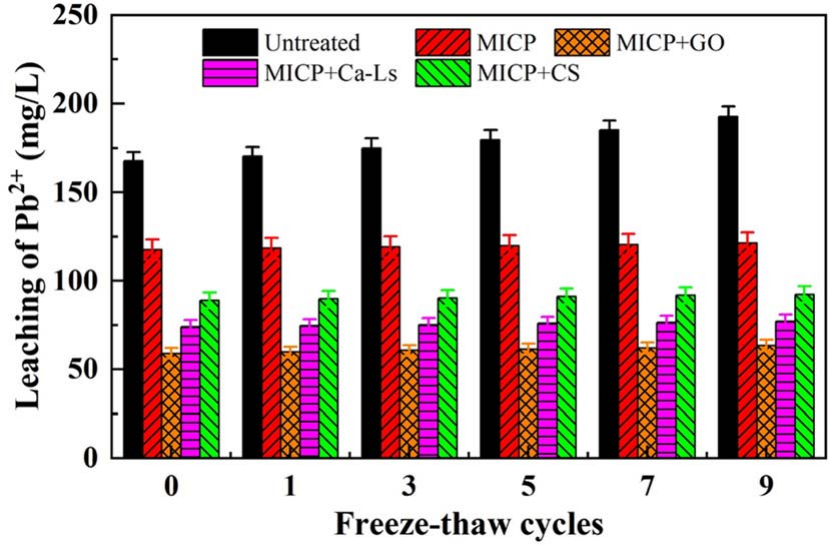
Relationship between leaching of Pb^2+^ and additives under freeze–thaw cycles.

Pb^2+^ leaching showed a complementary trend (see Fig. 8). In the absence of freeze–thawing, leachate concentrations were highest in untreated samples (about 170 mg L^−1^), followed by MICP (about 120 mg L^−1^), MICP + CS (about 90 mg L^−1^), MICP + Ca-Ls (about 70 mg L^−1^), and MICP + GO (about 55 mg L^−1^). These correspond to reductions of approximately 29, 47, 59 and 68 percent relative to untreated loess. Repeated freezing and thawing led to a gradual increase in leaching across all groups, with untreated soils rising to about 195 mg L^−1^ after nine cycles. In contrast, GO remained at only 68 mg L^−1^, Ca-Ls at about 80 mg L^−1^, CS at 92–95 mg L^−1^, and MICP at 118–122 mg L^−1^. Even after extended cycling, therefore, the GO group still exhibited about a 65 percent reduction compared with untreated loess, Ca-Ls maintained about 59 percent, CS about 51 percent, and MICP only about 38 percent. These findings highlight that additives not only enhanced the mechanical integrity of the treated loess but also significantly suppressed the mobility of Pb^2+^, with GO showing the most consistent and durable effect.

The co-evolution of strength loss and leaching increase can be attributed to microstructural changes induced by freeze–thaw cycling. Repeated ice formation and melting promoted volumetric expansion, crack initiation, and propagation, which disrupted CaCO_3_ cement bridges, reduced load-bearing capacity, and created additional pathways for solute migration. Additives modified both the rate and magnitude of these changes. GO, with its abundance of carboxyl, hydroxyl, and epoxy groups, enhanced Pb^2+^ adsorption and acted as a heterogeneous nucleation template, while its two-dimensional sheet structure likely reinforced interparticle contacts and hindered crack propagation. This translated into higher UCS values, more persistent alkalinity, and the lowest leaching concentrations. Ca-Ls provided similar, though somewhat weaker, benefits by improving cementation uniformity through polyanionic complexation and dispersion, and by modulating CaCO_3_ crystal morphology. In contrast, CS, as a cationic polysaccharide, appeared to compete with microbial and EPS surface charges, leading to weaker urease activity and lower initial precipitation. Nevertheless, its polymeric chains may have contributed to a composite mineral–organic network that buffered strength loss under freeze–thaw stress, even though its overall immobilization performance remained less effective than GO or Ca-Ls. Without additives, MICP produced more heterogeneous cementation, rendering the system particularly vulnerable to freeze–thaw damage, with the largest absolute strength loss and limited capacity to suppress Pb^2+^ release.

Taken together, these results show that additive-mediated regulation of microbial activity, porewater chemistry, and nucleation pathways governs both the mechanical resilience and geochemical stability of MICP-treated Pb-contaminated loess under freeze–thaw cycling. Maintaining a high degree of urea hydrolysis and a stable alkaline environment, coupled with interfacial reinforcement and templated carbonate precipitation, is critical for achieving both mechanical durability and chemical immobilization. Under the conditions of this study, GO provided the most favorable balance between strength retention and Pb^2+^ stabilization, followed by Ca-Ls, with CS offering moderate benefits, whereas untreated and additive-free MICP soils deteriorated substantially under cyclic freezing.

### 3.5 Disscussion

**Fig. 9:**
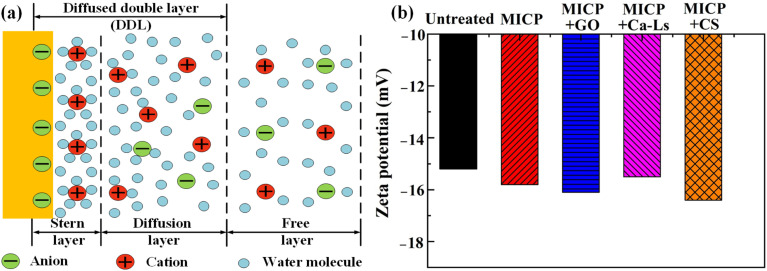
(a) Schematic illustration of the diffuse double layer, (b) variation of the zeta potential exposed to MICP technology and MICP technology modified by different additives.

The schematic represents the diffuse double layer (DDL) at the mineral–EPS interface (see Fig. 9). A negatively charged surface forms a compact Stern layer adjacent to the solid, while counter- and co-ions distribute in the surrounding diffuse layer; the zeta potential is defined at the hydrodynamic slipping plane within the DDL. Small changes in interfacial chemistry therefore translate into measurable shifts in zeta potential and, in turn, alter ion accumulation and nucleation near particle contacts. Untreated loess exhibited a zeta potential of about −14.5 to −15.0 mV. MICP without additives shifted the value to approximately −15.6 mV, consistent with deprotonation under alkaline conditions and the enrichment of negatively charged groups on cell walls and EPS during ureolysis. Additives produced distinct responses: MICP + GO measured approximately −16.0 mV, MICP + Ca-Ls approximately −15.3 to −15.5 mV, and MICP + CS approximately −16.2 to −16.4 mV. Thus, relative to untreated loess, the surface became more negative by roughly 0.6–1.5 mV after MICP, with the largest magnitudes for CS and GO and the smallest for Ca-Ls.

These shifts arise from additive-specific chemistries acting within the DDL. Graphene oxide introduces dense carboxyl, hydroxyl and epoxy groups that increase negative site density on adsorbed sheets and act as heterogeneous templates for CaCO_3_, which strengthens counter-ion (Ca^2+^, Pb^2+^) recruitment into the Stern layer and elevates local activity products in the diffuse layer. Calcium lignosulfonate contributes sulfonate and carboxylate groups but its intrinsic Ca^2+^ can partially screen charges through ionic bridging, yielding a slightly less negative potential than GO. Chitosan, although cationic in acid, is largely deprotonated in the alkaline MICP microenvironment; adsorbed CS presents neutral to weakly negative moieties capable of complexing Ca^2+^/Pb^2+^, producing a markedly negative zeta potential, yet polymer coatings can impose steric effects and dampen microbial dispersion and urease activity. Mechanistically, a more negative zeta potential within the DDL enhances counter-ion accumulation at the slipping plane, shortens the time to carbonate supersaturation, and increases the probability of heterogeneous nucleation. This pathway is strongest for GO, consistent with its higher fractions of carbonate-bound and residual Pb and superior UCS. Ca-Ls achieves slightly weaker electrostatics but improves spatial uniformity of precipitation *via* polyanionic complexation and dispersion. CS yields a very negative potential but moderates bio-nucleation efficiency, leading to only moderate gains in cementation and Pb stabilization. Collectively, the DDL analysis indicates an optimal window of surface charge: sufficiently negative to drive counter-ion enrichment and nucleation, yet not dominated by polymer adsorption that suppresses microbial activity. Under the present conditions, GO operates nearest this window, Ca-Ls trades electrostatic strength for uniformity, and CS emphasizes surface negativity at the expense of biological efficiency.

**Fig. 10:**
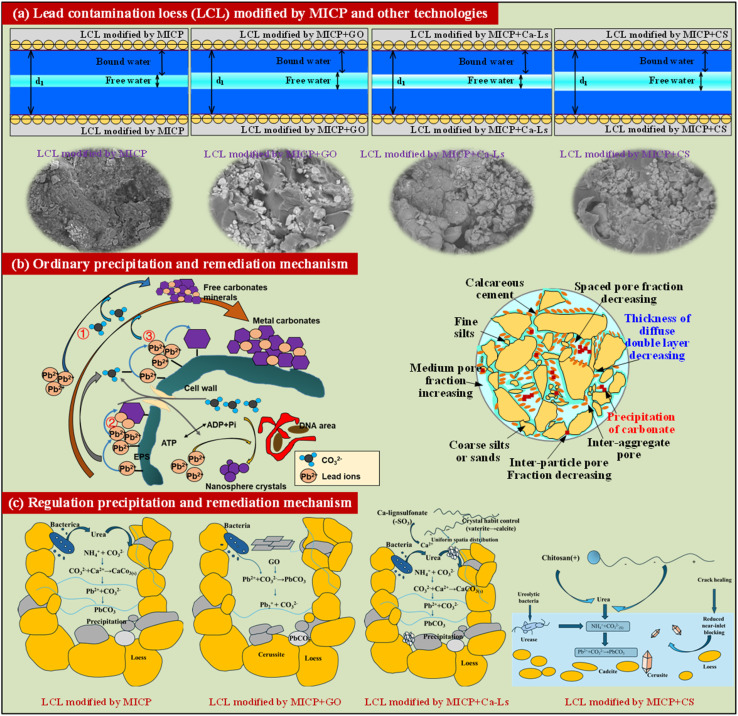
(a) Lead contaminated loess (LCL) modified by MICP and other technologies, (b) orfinary precipitation and remediation mechanism and (c) regulation precipitation and remediation mechanism.

The mechanistic schematic, combined with SEM observations, highlights the critical role of DDL regulation and carbonate precipitation pathways in determining the efficiency of MICP remediation of Pb-contaminated loess (see Fig. 10). In untreated MICP systems, bacterial ureolysis generates carbonate ions that interact with Ca^2+^ and Pb^2+^ within the pore network. SEM images reveal relatively heterogeneous precipitation, with discrete calcite clusters and incomplete filling of interparticle pores. The associated DDL remains comparatively thick, sustaining larger fractions of free water and wider pore channels, which weaken cementation and limit long-term Pb stabilization.

The incorporation of graphene oxide markedly alters this microenvironment. Its abundant oxygenated functional groups (–COOH, –OH, –O–) impart additional negative charge to particle and bacterial surfaces, leading to a contraction of the DDL and stronger electrostatic attraction of Ca^2+^ and Pb^2+^. SEM images confirm denser carbonate bridges and more continuous crystalline networks, which reduce pore connectivity and enhance encapsulation of Pb. This synergy between electrostatic regulation and templated nucleation explains why MICP + GO delivers both the highest strength and the most durable immobilization. The immobilization mechanism of Pb^2+^ within the loess matrix is likely governed by carbonate-related solidification rather than simple hydroxide precipitation. Under the alkaline microenvironment near the cathode and the abundance of Ca^2+^ and CO_3_^2−^ ions, Pb^2+^ can co-precipitate with CaCO_3_ or partially substitute Ca^2+^ within the carbonate lattice to form mixed Ca–Pb carbonates (Pb_*x*_Ca_1−*x*_CO_3_). Similar substitution and incorporation behaviors have been reported in previous studies,^1,2,22–24^ where lead immobilization proceeded through the formation of thermodynamically stable carbonate phases. Therefore, the stabilized Pb in this study is most plausibly associated with Ca–Pb composite carbonates rather than less stable Pb(OH)_2_ precipitates, consistent with the observed high immobilization efficiency.

Calcium lignosulfonate introduces polyanionic sulfonate and carboxyl groups that similarly contract the DDL by increasing the density of surface charges, while its dispersive action suppresses premature clogging and promotes more uniform crystal growth. SEM morphology shows relatively homogeneous carbonate precipitation along particle contacts, with finer crystals bridging aggregates. Although the magnitude of surface potential change is less than that induced by GO, Ca-Ls enhances spatial uniformity of cementation and reduces localized pore pathways for Pb migration.

In contrast, chitosan exhibits a distinct mechanism. Although its amino groups are protonated under acidic conditions, the alkaline environment of MICP reduces its cationic charge, allowing partial complexation with Pb^2+^ and Ca^2+^. The DDL is moderately compressed, but SEM images reveal less continuous carbonate networks and evidence of microcracks. Chitosan chains form a polymeric coating that hinders bacterial dispersion and urease activity, resulting in fewer nucleation sites and lower precipitation uniformity. While some pore filling occurs, the cementation is patchy, leaving residual pores that facilitate Pb mobility under external stress. The integration of DDL theory and SEM evidence indicates that additives act by tuning interfacial charge density, thereby controlling carbonate nucleation and growth patterns within the loess matrix. GO drives the strongest DDL contraction and most extensive crystal bridging, Ca-Ls balances moderate charge regulation with enhanced precipitation uniformity, and CS reduces overall efficiency by limiting microbial activity despite inducing a strongly negative surface potential. This mechanistic framework explains the observed differences in UCS retention and Pb leaching resistance under freeze–thaw cycles, and underscores that effective remediation requires optimizing both electrostatic regulation and microstructural consolidation.

The combined analysis of diffuse double layer (DDL) regulation and SEM imaging provides a coherent framework for understanding how different additives modulate MICP in Pb-contaminated loess. Graphene oxide contracts the DDL through its oxygenated functional groups, enriches Ca^2+^ and Pb^2+^ at particle interfaces, and promotes dense carbonate bridges, yielding superior strength and immobilization. Calcium lignosulfonate increases surface charge density while dispersing precipitates, enhancing spatial uniformity and long-term stability. Chitosan, although generating a strongly negative zeta potential, introduces polymer coatings that suppress microbial activity and produce patchy cementation, leading to suboptimal consolidation. These results highlight that the balance between electrostatic regulation and microstructural integration is decisive for the dual goals of mechanical reinforcement and Pb stabilization. Despite these advances, the present study faces limitations. First, zeta potential measurements capture average interfacial conditions but cannot fully resolve dynamic changes in the DDL during cyclic loading or long-term field conditions. Second, SEM evidence provides only two-dimensional snapshots, which may underestimate the complexity of carbonate distribution in three-dimensional pore networks. Third, the study focused on a single contaminant (Pb^2+^) and controlled laboratory conditions, leaving open questions about multi-metal interactions, competitive ion effects, and natural hydrogeochemical variability. Finally, freeze–thaw cycling was used as the primary environmental stressor, but other field-relevant perturbations, such as wet–dry cycling, chemical aging, and microbial succession, remain unexplored.

Future research should integrate *in situ* spectroscopic and tomographic techniques, such as X-ray CT, nano-FTIR and synchrotron-based mapping, to capture the spatiotemporal evolution of carbonate networks and contaminant immobilization at the microscale. Advanced molecular simulations could complement experimental work by quantifying ion transport and nucleation dynamics within additive-modified DDLs. Extending MICP remediation to multi-metal systems such as Pb–Cu and Pb–Zn under coupled mechanical and hydrochemical stressors would provide greater environmental relevance. In addition, the development of smart additives including engineered nanomaterials or bio-inspired polymers with tunable surface chemistry offers a promising direction to optimize both microbial activity and precipitation pathways. Finally, bridging laboratory findings with field-scale demonstrations will be essential to evaluate the durability, scalability and ecological safety of additive-assisted MICP for the remediation of contaminated soils.

## 4. Conclusions

This study investigated the effects of three representative additives, GO, Ca-Ls and CS, on the performance of MICP for stabilizing Pb-contaminated loess. The results integrated zeta potential characterization, SEM microstructural analysis, UCS and Pb^2+^ leaching tests under freeze–thaw cycles, providing a comprehensive understanding of additive–microbe–mineral interactions. The main conclusions from the results and discussion are as follows:

(a) GO introduced abundant oxygenated functional groups, contracted the diffuse double layer, and promoted dense carbonate bridging. It delivered the highest UCS (about 460 kPa initially, 350 kPa after nine cycles) and the lowest Pb^2+^ leaching (about 55–68 mg L^−1^), confirming its superior mechanical reinforcement and immobilization efficiency.

(b) Ca-Ls improved cementation uniformity through polyanionic complexation and dispersive effects. It showed moderate zeta potential shifts and stable UCS retention (∼330 kPa after nine cycles), with Pb^2+^ leaching maintained at about 70–80 mg L^−1^, indicating reliable stabilization under cyclic stress.

(c) CS induced strongly negative surface potentials but suppressed microbial activity, producing patchy precipitation and microcracking. Although strength retention was relatively high (82%), its absolute UCS and Pb^2+^ immobilization were weaker (90–95 mg L^−1^) than GO and Ca-Ls.

(d) The results demonstrate that additive regulation of the diffuse double layer and precipitation pathways is key to achieving durable MICP stabilization. Among the tested strategies, GO provided the most effective balance between strength and Pb^2+^ immobilization, Ca-Ls offered moderate but robust performance, and CS showed limited benefits.

## Author contributions

Jihua Gao: data curation, formal analysis, validation, software, writing – original draft. Wen-Le Hu: conceptualisation, methodology, writing – review & editing, supervision, funding acquisition. Pengli He: data curation, formal analysis, validation, software, writing – original draft. Longping Luo: data curation, formal analysis, validation, software, writing – original draft. Shixu Zhang: data curation, formal analysis, validation. Zifeng Hui: data curation, formal analysis, validation. Chongyang Zhang: conceptualisation, methodology, writing – review & editing. Kangwei Wang: conceptualisation, methodology, writing – review & editing. Rong Fan: conceptualisation, methodology, writing – review & editing.

## Conflicts of interest

The authors declare that they have no known competing financial interests or personal relationships that could have appeared to influence the work reported in this paper.

## Data Availability

The data used to support the findings of this study are included within the article.
